# Dry Fermented Sausages with Total Replacement of Fat by Extra Virgin Olive Oil Emulsion and Indigenous Lactic Acid Bacteria

**DOI:** 10.17113/ftb.59.03.21.7114

**Published:** 2021-09

**Authors:** Taxiarchoula Magra, Nikolaos Soultos, Chrysostomos Dovas, Ekaterini Papavergou, Thomai Lazou, Ilias Apostolakos, Georgia Dimitreli, Ioannis Ambrosiadis

**Affiliations:** 1 Department of Hygiene and Technology of Foods of Animal Origin, School of Veterinary Medicine, Faculty of Health Sciences, Aristotle University of Thessaloniki, 54124 Thessaloniki, Greece; 2 Diagnostic Laboratory, School of Veterinary Medicine, Faculty of Health Sciences, Aristotle University of Thessaloniki, 54627 Thessaloniki, Greece; 3 Central Research Laboratory for the Physical and Chemical Testing of Foods, Department of Food Science and Technology, International Hellenic University, P.O. Box 141, 57400 Thessaloniki, Greece

**Keywords:** fat substitute, probiotics, fermented meat products

## Abstract

**Research background:**

Formulations based on vegetable or fish oil and modifications in the production technology of dry fermented sausages have emerged in recent years aiming to achieve the desirable target of reducing the fat content of these meat products. However, previous efforts have confronted many difficulties, such as high mass loss and unacceptable appearance due to intensely wrinkled surfaces and case hardening. The objective of this study is to produce and evaluate dry fermented sausages by utilising a meat protein-olive oil emulsion as fat substitute and indigenous lactic acid bacteria (LAB) with probiotic properties isolated from traditional Greek meat products.

**Experimental approach:**

A novel formulation with extra virgin olive oil and turkey protein was developed to totally replace the conventionally added pork fat. Probiotic and safety characteristics of autochthonous LAB isolates from spontaneously fermented sausages were evaluated and three LAB isolates were finally selected as starter cultures. Physicochemical, microbiological and sensory analyses were carried out in all treatments (control, *Lactobacillus acidophilus*, *L. casei*, *L. sakei* and *Pediococcus pentosaceus*) during fermentation.

**Results and conclusions:**

Ready-to-eat sausages were found to be microbiologically stable. The olive oil-based formulation produced in this study generated a mosaic pattern visible in the sliced product simulating the fat in conventional fermented sausages and was regarded as an ideal fat substitute for the production of fermented sausages. An autochthonous isolate of *Lactobacillus casei* adapted the best to the final products as it was molecularly identified to be present in the highest counts among the LAB isolates used as starter cultures.

**Novelty and scientific contribution:**

Α novel and high-quality dry fermented meat product was produced by replacing the added pork fat with a fat substitute based on a meat protein-olive oil emulsion. Autochthonous LAB with *in vitro* probiotic properties could have a potential use in large-scale novel dry fermented sausage production. Such isolates could be used as starters in an effort to standardise the production process and retain the typical organoleptic and sensory characteristics. Moreover, isolates like *L. casei* 62 that survived in high counts in the final products can increase the safety of fermented sausages by competing not only with pathogens but also with the indigenous microbiota and could have a potential functional value for the consumer.

## Introduction

Dry fermented sausages are widely consumed meat products with special sensory characteristics such as exceptional taste and distinctive flavour (*1*). However, conventional dry fermented sausages cannot be classified as healthy foods according to the modern nutritional trends that praise the benefits of low-fat diets since their fat mass fraction is high (30.0-50.0 g fat per 100 g) and are rich in saturated fatty acids (*2*). High animal fat intake can contribute to the prevalence of the modern nutrition-related health problems such as heart disease, hypertension, obesity, diabetes and cancer (*3*). Therefore, WHO (*3*) and USDA (*4*) have proposed to limit the daily fat intake to less than 30.0% of total calories and to adapt a Mediterranean type of diet that includes higher consumption of olive oil that is rich in unsaturated fatty acids.

New formulations based on vegetable or fish oil and modifications in the production technology of dry fermented sausages have emerged in order to achieve the desirable target of reducing the fat content of these meat products (*2*, *5*). However, the anticipated reduction or replacement of fat content cannot be infinite in these products since fat plays an important technological role by contributing to the continuous loss of water and diffusion of moisture outwards, procedures with positive impact on the fermentation process. Moreover, fat contributes to various properties of the final product such as flavour, texture, mouthfeel, juiciness and lubricity (*6*). No data are available about achieving total replacement of the added animal fat in fermented sausages. Previous efforts towards reducing the fat content of dry fermented sausages confronted many difficulties. In particular, compared to their ordinary counterparts, dry fermented sausages with low fat content (more than 30.0% reduction) appeared harder, with higher mass loss and unacceptable appearance due to the intensely wrinkled surfaces and case hardening (*2*, *6*, *7*).

The use of starter cultures in dry sausage production is deemed necessary in order to control the manufacturing process, attain the standardised sensory properties and guarantee the microbiological safety of the final product. Starter cultures may exhibit *in vitro* probiotic properties such as growth at low pH values, tolerance of different concentrations of bile salts and antimicrobial activity against pathogens. Nevertheless, the *in vivo* functional value of viable (>6.0 log CFU/g) probiotic cultures in foodstuffs should entail clinically based evidence of specific beneficial effects to human health such as desirable modulation of intestinal flora, prevention of diarrhoea, improvement of constipation symptoms, prevention of allergies and reduction of plasma cholesterol levels (*8*, *9*). Commercial probiotic cultures of lactic acid bacteria (LAB) used as starter cultures in industrial production of dry fermented sausages are of milk product origin (*10*). However, non-commercial LAB with *in vitro* probiotic properties have been isolated from other sources, like infant and pig faeces and fermented vegetables, and used as starter cultures in dry fermented sausages resulting in high detectable populations in the final products (>6.0 log CFU/g), highlighting a potential functional value of such products (*9*-*11*). The isolation of LAB of meat product origin remains an issue of ongoing research since they are regarded more competitive and well-adjusted to the microenvironment of fermented sausages (*12*, *13*). The use of such LAB isolates as starter cultures with potential probiotic superiority compared to their commercially available counterparts could improve and optimise the sausage fermentation resulting in products with exceptional quality (*13*). LAB isolates with *in vitro* functional properties have been isolated from spontaneously fermented meat products for potential use as starter cultures in fermented sausage production (*12*, *14*). In particular, Ruiz-Moyano *et al*. (*11*) produced dry fermented sausages using a meat-origin *Pediococcus acidilactici* isolate with *in vitro* probiotic properties without reporting any notable modifications on the physicochemical and sensory quality of the final products.

This study aims to produce dry fermented sausages by combining a novel replacement of pork fat with extra virgin olive oil and the addition of indigenous starter cultures isolated from traditional meat products. For this purpose, an edible pork fat substitute with maximum extra virgin olive oil and minimum possible water content was produced in order to totally replace the added pork fat in dry fermented sausages. Another aim is to isolate, classify and examine the most important probiotic and safety characteristics of autochthonous LAB isolates from spontaneously fermented sausages in order to evaluate their utilisation as starters in fermented sausage production. The olive oil-based formulation and selected autochthonous LAB isolates were used for the production of dry fermented sausages and their sensory, microbiological and physicochemical parameters were evaluated.

## Materials and methods

### Production of edible pork fat substitute

In order to replace the added pork fat in dry fermented sausages with a substitute based on extra virgin olive oil, it was critical to ensure that the latter would be solid and stable at both 4 °C and room temperature, and resemble pork fat in terms of hardness, colour and appearance. Therefore, olive oil was solidified in a formulation with turkey lean meat as an emulsifier. Turkey breast meat was selected as a source of natural proteins that would provide a light colour to the fat replacer, the anticipated technological characteristics, such as solidification, and concurrently contribute to the desired decrease of the fat content in the final product. The extra virgin olive oil formulations were heat-treated (65 °C/40 min) prior to use in order to achieve the denaturation of proteins and subsequent desired solidification of the formulation.

Different formulations were produced and evaluated during a preliminary research (data not shown). The formulation (in 100 g) with the best appearance and hardness consisted of 41.0 g turkey meat (4 °C), 16.0 g ice water, 2.5 g salt, 0.5 g phosphates (Sigma-Aldrich, Merck, St. Quentin Fallavier, France) and 0.2 g white pepper. All ingredients were chopped (Kilia 30L cutter; Kilia Fleischinenfabric, Kiel, Germany) until the temperature of the mixture reached 2 °C. Then, olive oil was added up to the final mass fraction of 35.0 g/100 g and mixed continuously for thorough integration. Mixing was completed after the addition of 4.7 g per 100 g potato starch (Sigma-Aldrich, Merck). The final mixture (temperature 12-14 °C) was stuffed into 40 mm diameter polyethylene-polyamide casings (RS Baby 3000 vacuum stuffer; Risco, Vicenza, Italy) and subsequently solidified with pasteurisation at 65 °C for 40 min. The stable formulations (stored at -18 °C) were added to the sausage mixture at the beginning of the cutting.

### Isolation and identification of indigenous LAB from meat products

Indigenous LAB present as autochthonous microbiota without any addition of starter cultures in Greek traditional dry fermented sausages (*N*=16) were isolated according to the ISO 15214:1998 method (*15*). In brief, 20 g of aseptically sliced sausage were homogenised with 180 mL peptone water (PW, LAB M, Lancashire, UK) in a Stomacher® 400 laboratory blender (Seward Medical, London, UK) followed by tenfold serial dilutions, pour plate inoculation of de Man, Rogosa and Sharpe agar (MRS agar, LAB M) and incubation at 30 °C for 72 h. In total, 32 out of 160 randomly selected isolates from the MRSA plates were initially characterised as LAB based on positive Gram stain (Sigma-Aldrich, Merck) and negative catalase and oxidase (Sigma-Aldrich, Merck) activity. A method previously described by Lazou *et al*. (*16*) was used for the extraction of genomic DNA of these 32 LAB isolates prior to their molecular identification. In brief, one loop of cells of each isolate was mixed with 100 μL of lysis buffer I (50 mM Tris-HCl, 50 mM EDTA, 4 M guanidine hydrochloride (GuHCl), 10 mM CaCl_2_, 1% Triton X-100 and 2% *N*-lauroyl-sarcosine, pH=7.5; Merck, Darmstadt, Germany) and 25 μL proteinase K (0.56 mg; New England Biolabs, Ipswich, MA, USA), and the mixtures were incubated at 56 °C for one hour. Then, 250 μL of lysis buffer II (50 mM Tris-HCl, 25 mM EDTA, 8 M GuHCl, 3% Triton X-100 and 3% *N*-lauroyl-sarcosine, pH=6.3; Merck) were added and the mixtures were incubated at 70 °C for 10 min. Absolute ethanol (250 μL; Merck) was added to the lysates, and each mixture was passed through a silica column (FT-2.0; G. Kisker GbR, Steinfurt, Germany) by centrifugation (8000×*g*; Thermo Scientific™ Sorvall™ Legend™ Micro 17 Microcentrifuge, Fisher Scientific, Suwanee, GA, USA). Columns were washed three times, once with wash buffer I (25 mM Tris-HCl, 4 M GuHCl, and 50% ethanol, pH=6.6; Merck), and then twice with wash buffer II (10 mM Tris-HCl, 0.1 M NaCl, and 80% ethanol, pH=6.6; Merck), followed by elution with 100 μL TE buffer composed of 10 mM Tris-HCl and 1 mM EDTA, pH=8.1 (Merck).

For the molecular identification of indigenous LAB isolates, a previously described polymerase chain reaction (PCR) assay targeting the 16s-23s rRNA intergenic spacer region was applied (*17*). Briefly, the 16S-23S intergenic spacer region (ISR) from these isolates was amplified using primers that annealed to conserved regions of the 16S and 23S genes. These primers were 16S/p2 (5’-CTTGTACACACCGCCCGTC-3’) and 23S/p10 (5’-CCTTTCCCTCACGG-TACTG-3’), which anneal to positions 1392 to 1410 of the 16S rRNA gene and to positions 713 to 731 of the 23S rRNA gene (*Lactobacillus salivarius*, GenBank accession number CP002034), respectively (*18*). The obtained PCR product corresponded to the complete 16S-23S ribosomal ISR and parts of the flanking rDNAs. PCR mixtures contained 2 μL purified DNA, 2 μL of 10× polymerase buffer (Boehringer Mannheim GmbH, Mannheim, Germany), 100 μM each deoxynucleoside triphosphate, 400 μL each primer, 200 μL MgCl_2_ (Life Technologies, Invitrogen, Bleiswijk, The Netherlands), 1.5 U of Expand High Fidelity PCR System (Boehringer Mannheim) DNA polymerase and nuclease-free water up to a total volume of 20 μL per reaction. DNA amplification began with a pre-incubation step at 94 °C for 7 min, followed by 40 cycles of the following thermal cycling conditions: 95 °C for 30 s, 56 °C for 30 s, and 72 °C for 40 s. The final extension step included 3-minute incubation at 72 °C. The amplification products were electrophoresed in a 1.5% agarose gel containing 0.5% ethidium bromide and visualised by UV illumination. Subsequently, the smallest spacer region PCR product (approx. 800-900 bp) of each isolate was excised from the agarose gel and extracted using a NucleoSpin Extract II kit (Macherey-Nagel, Duren, Germany). All strands were sequenced using the PCR primers 16S/p2 and 23S/p10 and internal primers 16S/p4 (5’-GCTGGATCACCTCCTTTCT-3’ and 23S/p7 (5’-TGCAGGTACTTAGATGTTTCAGTTC-3’). Sequencing was performed by utilising a BigDye Terminator v3.0 ready reaction cycler sequencer kit (Applied Biosystems Inc., Foster City, CA, USA) according to the manufacturer’s instructions. The obtained small intergenic spacer region sequences were compared to sequences from type cultures and other *Lactobacillus* strains held in GenBank (*18*) using the BLAST algorithm (*19*).

### Probiotic properties and safety characteristics

Each of the following procedures for assessing some of the basic probiotic properties and safety characteristics of the identified LAB isolates was conducted in triplicates.

#### Acid resistance and bile tolerance

Acid resistance and bile tolerance of the isolated LAB (*N*=32) were examined in modified media adjusted to obtain a final pH=2.0 and 3.0 or containing 0.3% (*m*/*V*) oxgall (Oxoid, Hampshire, UK), respectively, according to the method described by Gu *et al*. (*20*).

#### Biogenic amine production

The undesired potential tyramine (TY) and histamine (HI) forming capacity of the 32 LAB isolates was determined by high-performance liquid chromatography with a gradient elution system (Waters 470 HPLC system, Waters, Milford, MA, USA) as described by Hernández-Jover *et al*. (*21*). Shortly, the HPLC consisted of a system controller pump, an autosampler, a reagent delivery modul (RDM) postcolumn reaction equipment and a 470 spectrofluorometric detector. A coil 200 cm long and stainless-steel tubing were used to connect the detector. The separation was performed on a C18 column, 4 μm particle size, with a matching guard cartridge.

#### Antibiotic susceptibility

The disk-diffusion method according to the Clinical and Laboratory Standards Institute guidelines (*22*) was applied to assess the antibiotic susceptibility profile of eight LAB isolates. They were selected due to their resistance to low pH values and bile salts and the inability to produce biogenic amines. The following 18 antimicrobial agents (amount per disk; BD BBL™ Sensi-Disc™, Sparks, MD, USA) were tested: amoxicillin (25 mg), ampicillin (10 mg), cefotaxime (30 mg), cephalothin (30 mg), chloramphenicol (30 mg), ciprofloxacin (5 mg), clindamycin (2 mg), erythromycin (15 mg), gentamycin (10 mg), kanamycin (30 mg), nalidixic acid (30 mg), neomycin (30 mg), penicillin (10 U), streptomycin (10 mg), sulfamethoxazole (23.75 mg) and trimethoprim (1.25 mg), tetracycline (30 mg) and vancomycin (30 mg) (*20*, *23*).

#### Antimicrobial activity against pathogens

Based on the results of the aforementioned analyses, the antimicrobial activity of five (*N*=5) selected LAB isolates was tested against the following pathogenic strains: *Staphylococcus aureus* F137, *Staphylococcus aureus* F264, *Escherichia coli* ATCC 11303, *Salmonella* Typhimurium ATCC 14028, *Listeria monocytogenes* ATCC 7644 and *Bacillus cereus* ATCC 14579. These strains were grown overnight in tryptone soya yeast extract broth (Sigma-Aldrich, Merck) at 37 °C and a diluted suspension containing approx. 6.0 log CFU/mL was spread onto a tryptone soya yeast extract agar (Sigma-Aldrich, Merck) plate. From overnight cultures of 6.0 log CFU/mL of the tested LAB isolates, a volume of 500 μL was added to sterile paper disks (13 mm diameter), left to dry and then placed onto the previously inoculated plates that were incubated aerobically at 37 °C for 24 h. Subsequently, inhibition zone diameters were measured with a precision calliper (Traceable™ Digital Caliper, Fisher Scientific, Loughborough, UK). LAB isolates with inhibition zones of 1.30, 1.30-1.80 and >1.80 cm were classified as isolates with absent (-), strong (++) and very strong (+++) inhibition, respectively (*23*).

### Production of dry fermented sausages with olive oil and LAB with probiotic properties

All dry fermented sausages were produced with total replacement of pork fat using the novel extra virgin olive oil-based formulation. The lean pork and beef meat were trimmed from visible fat and connective tissue. The recipe (per 100 g) consisted of 48.0 g lean pork meat (max 5.0 g fat), 22.0 g lean beef meat (max 7.0 g fat), 25.0 g pork fat substitute, 2.1 g curing salt (2.085 g NaCl and 0.015 g NaNO_2_), 2.0 g citrus dietary fibre (Fiberstar Citri-Fi, River Falls, WI, USA), 0.8 g dextrose and 0.1 g white pepper. The frozen pork meat (<-18 °C) and the extra virgin olive oil formulation (<-18 °C) were chopped at low speed followed by the addition of the minced beef, additives and finally salt with nitrites. The final mixture was chopped until the desired particle size (3-5 mm) was attained and subsequently stuffed into 40.0 mm diameter permeable cellulose casings (RS Baby 3000 vacuum stuffer, Risco).

One commercial probiotic culture (*Lactobacillus acidophilus* Alce LMGP 21381; LA) and three indigenous LAB isolates isolated during this study that combined the most desirable probiotic properties (*Lactobacillus casei* 62, *Lactobacillus sakei* 65 and *Pediococcus pentosaceus* 156) were selected as starter cultures for the production of equal number of fermented sausages during three independent experiments. Sausages produced in each experiment without the addition of a LAB culture served as the control (C) treatment.

Fermentation and ripening took place in a controlled-climate unit for 21 days in total. During the first six days (fermentation period), the temperature was gradually reduced from 22 to 15-16 °C, the relative humidity from 95.0 to 85.0% and the air velocity from 0.7 to 0.5 m/s. Throughout the following days (ripening period), the temperature was set at 15 °C, the relative humidity at 82.0-84.0% and the air velocity at 0.05-0.10 m/s. Sausages produced in each treatment were sampled in duplicate for physicochemical (1st, 3rd, 7th, 14th and 21st day of production), chemical (1st and 21st day of production) and microbiological analyses (1st, 3rd, 7th, 14th and 21st day of production). Sensory evaluation of all samples was performed on the 21st day of production. Lactic acid bacteria were also enumerated after 4 and 6 months of storage at 4 °C.

### Physicochemical analyses

Mass loss ratios (PCB 1000-2; Kern & Sohn GmbH, Balingen, Germany), pH values (WTW microprocessor pH-meter, WTW GmbH, Weinheim, Germany) and water activity (AQUA LAB Mod. CX-2, Decagon Devices Inc., Pullman, WA, USA) of produced sausages were measured (*24*).

### Chemical analyses

Moisture was determined by drying at (102±2) °C in a drying oven overnight (Heratherm™ General Protocol Ovens, Thermo Fisher Scientific, Bedford, MA, USA) to a constant mass, according to AOAC method 950.46 (*24*). Crude fat content was determined by extracting the fat from the dried sample using petroleum ether (Sigma-Aldrich, Merck) with a Soxtherm 2000 device (S306 AK model; Gerhardt, Königswinter, Germany) according to AOAC method 991.36 (*25*). Total nitrogen was determined in a Kjeldahltherm device (KB Digestion Systems model, Gerhardt) according to AOAC method 928.08 (*26*). The sample was digested with concentrated sulphuric acid (Sigma-Aldrich, Merck) and organic nitrogen was converted into ammonium sulphate. Then, sodium hydroxide (Sigma-Aldrich, Merck) was added in excess, ammonia was released by water steam distillation and trapped in a solution of boric acid (Sigma-Aldrich, Merck). Titration with acid solution (H_2_SO_4_) was done and the nitrogen content was calculated. Ash content of the samples was determined in an amount of dried, defatted sample. Ashing was carried out at 525 °C overnight (M110 Muffle Furnaces; Thermo Fisher Scientific). All chemical analyses for the evaluation of dietary value were performed in duplicate.

Lipid oxidation was determined by a selective third-order derivative spectrophotometric method (*27*). A rapid method for measuring malondialdehyde as a marker of lipid peroxidation in sausage samples was used. Samples were homogenised with trichloroacetic acid, hexane and butylated hydroxytoluene (all from Sigma-Aldrich, Merck) and then centrifuged (8000×*g*). Malondialdehyde was quantified based on the third derivative absorption spectrum of the pink complex formed, after reaction with thiobarbituric acid reagent (Sigma-Aldrich, Merck).

### Microbiological analyses

The following microbiological parameters were enumerated in the produced fermented sausages using tenfold serial dilutions, as described previously, followed by inoculation and incubation of the selective media as indicated in the corresponding ISO methods: LAB (*15*), coagulase-positive staphylococci (*28*), Enterobacteriaceae (*29*) and yeasts and moulds (*30*, *31*). *Listeria monocytogenes* was detected on the 1st and 21st day of production (*32*). A previously described multiplex polymerase chain reaction (PCR) assay (*33*) was used to molecularly identify five randomly selected presumptive *L. monocytogenes* colonies originally grown on Agar Listeria according to Ottaviani and Agosti (ALOA; Biolife, Milan, Italy) plates (*34*). This assay utilises a combination of genus- and species-specific primers and generates three possible band types indicative of bacterial, *Listeria* spp. and *L. monocytogenes* genomic DNA, respectively.

### Real-time PCR primers and conditions

DNA was extracted (*10*) from all the countable (25-250) LAB colonies isolated on MRS agar plates from the produced fermented sausages at the beginning (day zero) and the end of production (21st day) as well as after four and six months of storage at 4 °C. A real-time PCR assay was applied in order to identify the percentage of the aforementioned LAB colonies formed by the starter culture isolate used in each of the three corresponding treatments and to detect the potential existence of populations of the same species as the starter among the autochthonous microbiota of the control treatment. Since the overall acceptability of the fermented sausages was unknown at the beginning of their production, the real-time PCR assay was performed for LAB colonies originating only from treatments that received positive sensory evaluation. Therefore, LAB colonies isolated from the treatment with *P. pentosaceus* were excluded from molecular identification due to the observed sensory deficiencies during the evaluation of the final products. Primers targeting the conserved functional *tuf* gene for the elongation factor Tu (*35*) were designed for the specific detection of isolates belonging to the species *L. acidophilus*, *L. casei* and *L. sakei*. Each primer pair was designed after multiple alignments of available *tuf* gene sequences using the molecular evolutionary genetics analysist (MEGA) v. 6 software (*36*) and checked for specificity using the basic local alignment search tool (BLAST) algorithm (*19*). The specificity of each pair of primers was confirmed by applying respective real-time PCR assays using DNA templates from all three used *Lactobacillus* species (*L. acidophilus*, *L. casei* and *L. sakei*) (Table 1). The real-time PCR reactions and cycling conditions were performed in a CFX96 Touch™ real-time PCR detection system (Bio-Rad, Hercules, CA, USA). Amplification reactions were run in a total volume of 20 μL using 2 μL of DNA template. The optimal reaction conditions for real-time PCR were as follows: 3 U of HotStartTaq Plus DNA polymerase (Qiagen, Copenhagen, Denmark), 1× PCR buffer (Qiagen), 2 mmol/L MgCl_2_ (Qiagen), 0.2 μmol/L of each primer (Qiagen), 0.2 mmol/L dNTPs mix (Qiagen), 1× DNA-specific fluorescent dye EvaGreen® (Biotium, Hayward, CA, USA). Cycling conditions included a preliminary denaturation step at 94 °C for 15 min, followed by 45 cycles at 95 °C for 30 s, 60 °C for 30 s and 72 °C for 10 s. Succeeding amplification, a melting curve was analysed to confirm the correct amplification product by its specific melting temperature at (70-92±0.2) °C/5 s. All samples were run in triplicate and cycle threshold (Ct) values <38 were regarded as positive results.

**Table 1:** Primers used for the molecular identification of lactic acid bacteria (LAB) isolates with real-time polymerase chain reaction (PCR)

**Target species**	Sequence (5’–3’)	Name	Target gene	Product size/bp
*L. acidophilus*	ACAAGGAAGCTCAAGACCAAATCATG	LacF	*tuf*	238
	TCCAAACCAGTAACAACTGACTTAAGA	LacR		
*L. sakei*	CTGACGACGTTAAAGCTGTTGAAG	LSakF		224
	AACAGTTGTCTTAGCAATTTCTTCCT	LSakR		
*L. casei*	CCCTTGAAGGCGATCCAGAACA	LCaF		238
	ACGGTAGACTTGATAACATCTGGCT	LCaR		

### Sensory evaluation

A trained panel of individuals working in the academia and in the food industry (*N*=15) evaluated the overall acceptability of dry fermented sausages according to a 9-point hedonic scale (9=like extremely, 1=dislike extremely) (*37*). The sensory panel consisted of seven trained female panellists aged between 35 and 60 and eight male panellists aged between 40 and 67. All panellists had more than two years’ experience in the sensory assessment of meat and meat products. The evaluation of the overall acceptability of dry fermented sausages included the appearance of the cut, colour, cohesiveness, hardness, flavour and overall acceptance (*38*). All sausages were evaluated on the same day. Three replicates of each sample were evaluated. The sausage order was randomised and the sausages were served in the same order to all panellists. The panellists had maximum two bites of each sample. Between samples, the panellists were asked to cleanse the palate by drinking water and eating a piece of bread.

### Textural evaluation

An Instron Universal Testing Machine model 1140 (Instron Ltd., Wycombe, UK) was used for the textural measurements. It was equipped with a load cell of 0–500 and 0–50 kg for texture profile analysis (TPA) and the cut tests, respectively. The crosshead speed was set to 80 mm/min for both tests. Cylindrical samples of 23 mm in diameter and 21.5 mm in height were generated. The sausage samples were equilibrated for about an hour at room temperature ((20±1) °C) before testing and their textural properties were evaluated by the method of TPA. The samples were uniaxially compressed at room temperature to 80% of their original height and each one was subjected to two subsequent cycles (bites) of compression–decompression. Hardness 1 (H_1_) and 2 (H_2_), work done on the sample during the first bite (A_1_) and on the second bite (A_2_), cohesiveness (A_2_/A_1_), springiness (S_2_), gumminess (G) and chewiness (K) were calculated from the obtained profiles using the MathCAD software (*39*). TPA test was conducted in triplicate for each of the three replications (*40*). The cut test was also applied to the samples using a knife attached to the head of Instron tool (knife) that cut the samples in two pieces. The properties determined from the obtained curves were the force of cut (F) (the first peak of the curve indicating that the sample starts to cut) and the work done on the sample until the beggining of cutting (A) (*40*).

### Colour measurement

Colour measurements were carried out with a Hunter Lab, model Labscan 5000 spectrocolorimeter (Hunter Associates Laboratory, Inc., Reston, VA, USA) using a 10-mm port size, illuminant D65 and a 10° standard observer. CIELAB L*, a* and b* values were determined as indicators of lightness, redness and yellowness. Five measurements were made on the cross-section of three (5 cm long) pieces of sausage (*41*).

### Statistical analysis

A general linear model was applied to evaluate the statistical significance (p*<*0.05) of the differences observed between the starter LAB cultures regarding all the tested characteristics of the final products using the Minitab 17 Statistical Software (*42*). Tukey’s test was applied to compare the average scores that were statistically different at a confidence level of 5.0% (*42*). Moreover, PanelCheck (v. 1.4.0, Nofima) was used for a two-way analysis of variance for the results of sensory evaluation by panellists (*43*).

## Results and discussion

### Isolation of LAB from meat products

In the present study, the comparison of the sequenced 16s-23s rRNA gene boundary regions (GenBank accession numbers: MT431413 and MT431419) obtained from the LAB isolates with those held in the GenBank database enabled the identification of *Lactobacillus sakei* (*N*=14), *Lactobacillus casei* (*N*=4), *Lactobacillus plantarum* (*N*=2), *Lactobacillus brevis* (*N*=2), *Lactobacillus acidilactici* (*N*=2) and *Pediococcus pentosaceus* (*N*=8). These LAB were isolated from traditional Lefkadas sausages and Suzuk type dry fermented sausages, which were originally chosen because they are produced by LAB adapted to their microenvironment without the addition of any commercial starter cultures. LAB that have been isolated from fermented sausages with different production technologies and without the addition of starter culture usually belong to the genera *Lactobacillus, Weissella, Leuconostoc, Pediococcus, Enterococcus* and *Lactococcus* (*44*). Among them, lactobacilli are most frequently isolated and, in particular the species *Lactobacillus sakei, Lactobacillus curvatus* and *Lactobacillus plantarum* (*44*). Our results indicate that LAB were successfully isolated from both sujuk type and Lefkadas sausages despite their low pH values (<4.6) and high content of spices that usually inhibit LAB growth.

### Properties of LAB isolates

Acid and bile salt resistance are regarded two of the most important probiotic properties (*45*). Isolates of *Lactobacillus casei* (*N*=2), *L. sakei* (*N*=4) and *P. pentosaceus* (*N*=2) were able to grow at low pH values (<3.0) along with the presence of bile salts, contrary to the rest of the tested LAB isolates (*N*=24) that were unable to grow under these conditions.

None of the tested isolates (*N*=32) produced histamine, whereas one isolate of each *L. plantarum, P. acidilactici* and *L. brevis* produced tyramine (1755, 1577 and 1581 μg/mL of broth, respectively; data not shown). Tyramine and histamine are biologically active biogenic amines produced by the microbial decarboxylation of tyrosine and histidine, respectively. Their excessive consumption can cause nervous, gastric and intestinal or blood pressure problems and, consequently, increased attention is focused on their occurrence in foods (*46*). Presence of precursors (free amino acids), microorganisms with amino acid decarboxylase activity and favourable conditions for growth and decarboxylation are prerequisites for the production of biogenic amines. The extensive proteolysis during ripening in dry fermented sausages can provide such precursors for subsequent decarboxylation by microorganisms. The latter are either introduced in dry sausages as starter cultures or are part of the autochthonous microbiota. Indeed, Bover-Cid and Holzapfel (*47*) have reported the isolation of various LAB from fermented sausages with amino acid decarboxylating activity including several *L. curvatus, L. casei* and *L. plantarum* isolates. In this study, all isolates of *Lactobacillus casei, L. sakei* and *P. pentosaceus* did not produce biogenic amines and thus were deemed safe to be used in fermented sausage production.

Lactic acid bacteria have a long history of traditional use in fermented food technology. However, when harbouring transferable antibiotic resistance genes, they may serve as effective vehicles for antibiotic resistance transmission to other LAB or human pathogens, thus raising significant public health concerns. In recent years, increased focus has been given to foodstuffs as vehicles of antibiotic resistance genes (*48*, *49*). To prevent the undesirable transfer of resistance to endogenous bacteria, probiotics should carry the necessary resistance in terms of surviving in the presence of non-critically important antibiotics in the human intestinal microenvironment (*13*). Although special purpose probiotics for use in combination with antibiotics have been developed through the introduction of multiple resistance genes to their bacterial cells, probiotics should generally not carry more resistance than it is required for a specific purpose (*49*). Nevertheless, LAB with susceptibility to all antibiotic categories have not been isolated as yet (*50*). In this study, only eight LAB isolates were able to grow at low pH values and in the presence of bile salts, and did not produce biogenic amines and, therefore, were selected to be further tested for their antibiotic resistance against 18 antibiotics. These isolates were found to be resistant to ampicillin, kanamycin, vancomycin and sulfamethoxazole and susceptible to streptomycin, gentamycin, tetracycline, chloramphenicol, cephalothin, clindamycin, erythromycin and cefotaxime (data not shown). These findings are in accordance with the results of Clementi and Aquilanti (*51*). Only five out of these eight isolates were selected for further investigation, based on their susceptibility to ciprofloxacin and nalidixic acid and due to the fact that the top three critically important categories of antimicrobials for public health are macrolides, 3rd-4th generation cephalosporins and quinolones (*50*). More precisely, *L. casei* (*N*=1), *L. sakei* (*N*=3) and *P. pentosaceus* (*N*=1) isolates exhibited resistance to fewer antibiotics than the rest of the examined LAB isolates and, therefore, were further selected to test their antimicrobial activity against six food-related pathogens.

Finally, only the *L. sakei* isolates displayed antimicrobial activity against all tested pathogens. *L. casei* exhibited antimicrobial activity against *S.* Typhimurium and *B. cereus*, whereas *P. pentosaceus* showed moderate antimicrobial activity against all the examined pathogen isolates. Therefore, *L. casei* (*N*=1), *L. sakei* (*N*=1) and *P. pentosaceus* (*N*=1), which were isolated, molecularly identified and evaluated for their *in vitro* probiotic properties in this study, displayed desirable characteristics and were finally selected to be utilised as starters for the production of fermented sausages.

### Properties of dry fermented sausages with olive oil and probiotic LAB

#### Properties of fermented sausages with fat substitute

**Fig. S1:**
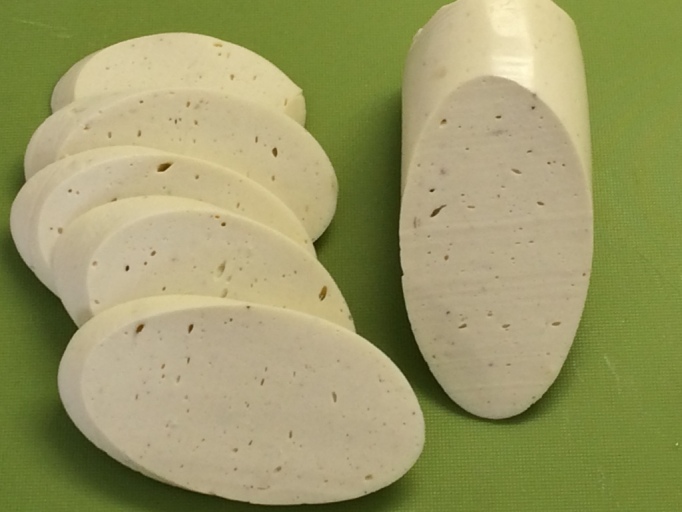
Appearance of the extra virgin olive oil and turkey meat formulation (solidified with pasteurization at 65 °C for 40 min) that was used as pork fat substitute in this study

The aim of this study was to produce ‘healthier’ fermented sausages. Pork fat was totally replaced by a substitute of extra virgin olive oil and turkey proteins. Extra virgin olive oil was chosen because of its exceptional dietary value, but it had to be solidified in order to avoid technological issues. Moreover, this substitute had to be of light colour in order to replace the pork fat and the final product to have an acceptable appearance for the consumers. By emulsifying olive oil with turkey proteins, the above aims were fulfilled (Fig. S1).

**Fig. S2:**
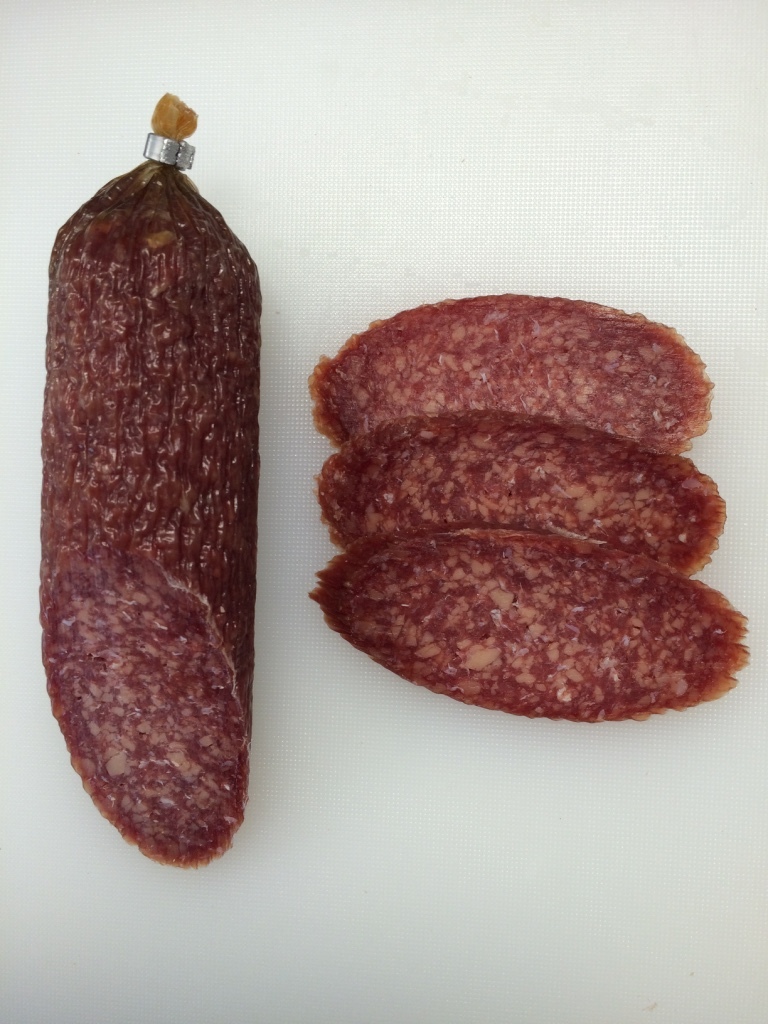
Appearance of the cut and overall appearance of dry fermented sausages produced in this study with extra virgin olive oil and *L. casei* 62

The olive oil-based formulation developed in this study replaced successfully the traditionally added pork fat in dry fermented sausages. The pasteurisation of the formulation effectively stabilised the extra virgin olive oil in the mass of the denaturised turkey proteins. Consequently, defects such as irregular mass loss, surface wrinkling or case hardening were not observed in the final products of this study in contrast to previously reported results regarding efforts of reducing or replacing the pork fat (*2*, *6*, *7*). Sausages were produced according to the traditional technologies with pork and bovine meat and fermented for 21 days, during which the mass loss was up to 30% (data not shown) in order to have comparable results. After the fermentation, sausages were pathogen-free and ready for consumption. During sensory evaluation, they obtained high scores for all sensory properties, such as flavour, firmness, appearance and overall acceptance. The emulsion of olive oil, forming a mosaic pattern, was visible in the sliced product in the same way that fat is visible in the conventional fermented sausages (Fig. S2).

#### Physicochemical properties of fermented sausages with fat substitute

Mass loss was approx. 30 g/100 g on the 21st day of production regarding all treatments. The addition of LAB cultures did not affect significantly the mass loss of sausages during the production compared to the control (p=0.497-0.856). However, after the 14th day of production, LAΒ and control treatments exhibited lower mass loss than the rest of the inoculated treatments. There were no statistically significant differences in the pH values (p=0.168-0.848) and water activity (p=0.389-0.832) throughout the production among the different treatments and both parameters decreased sufficiently, thus, ensuring the microbiological stability and safety of the final products. All four tested LAB cultures proved successful candidates for use as starter cultures, as they fermented the available sugars and produced lactic acid, causing the reduction of the pH value. They ensured the evolution of the fermentation and, therefore, highlighted their prospective application in large-scale sausage production (*45*, *52*, *53*).

#### Chemical parameters

**Fig. 1:**
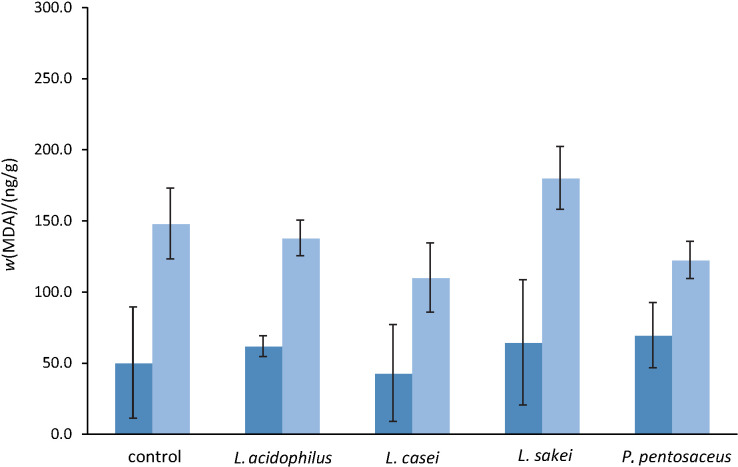
Lipid oxidation expressed as mass fraction of malondialdehyde (MDA) in dry fermented sausages with extra virgin olive oil and probiotic lactic acid bacteria (LAB) on the first (dark blue) and 21st (light blue) day of production in three independent experiments. Values are expressed as mean±standard error

The moisture mass fraction decreased significantly in all treatments (p<0.001) from the beginning to the 21st day of production, leading to the significant (p<0.001) increase in protein, fat and ash mass fraction (Table 2). Nonetheless, the addition of the starter culture did not influence significantly, among the different treatments, the moisture (p=0.297), ash (p=0.233), fat (p=0.654) and protein (p=0.223) mass fractions in the final products. It was logical to assume that the mass fraction (g/100 g) of monounsaturated fatty acids increased and of saturated fatty acids and cholesterol decreased compared to conventional fermented sausages since the olive oil-based formulation totally replaced the traditionally added pork fat and, therefore, no relevant measurements were carried out. The presence of extra virgin olive oil instead of pork fat enhances the beneficial effects of the sausages produced in this study and is in accordance with the principles of the Mediterranean diet that promotes the consumption of unsaturated fatty acids (*54*). The addition of the LAB cultures did not affect significantly the oxidation of fatty acids during the fermentation and ripening up to the 21st day of the production (p=0.501-0.663) among all treatments (Fig. 1). However, a statistically significant increase of malondialdehyde values in all treatments was observed (p<0.001), but without the detection of any rancid taste during the sensory evaluation of the final products.

**Table 2:** Moisture, ash, fat and protein mass fractions in dry fermented sausages with extra virgin olive oil and probiotic lactic acid bacteria (LAB) on the first and 21st day of production in three independent experiments

Treatment	*t*=0 day				*t*=21 day			
Moisture	Ash	Fat	Proteins	Moisture	Ash	Fat	Proteins
	*w*/%							
C	(64.2±0.7)^Αa^	(3.6±0.1)^Ab^	(10.7±0.6)^Ab^	(18.5±0.1)^Αb^	(51.5±1.2)^Αb^	(4.6±0.1)^Aa^	(14.8±1.2)^Aa^	(26.3±1.2)^Αa^
LA	(65.0±0.2)^Aa^	(3.4±0.1)^Αb^	(10.1±0.1)^Ab^	(18.5±0.1)^Αb^	(52.0±1.6)^Ab^	(4.4±0.1)^Aa^	(14.6±1.1)^Αa^	(26.3±1.1)^Aa^
LC	(64.3±0.4)^Aa^	(3.4±0.2)^Αb^	(10.8±0.2)^Αb^	(18.4±0.2)^Αb^	(51.5±1.1)^Ab^	(4.4±0.1)^Aa^	(14.5±1.5)^Αa^	(26.6±1.1)^Αa^
LS	(64.8±0.6)^Aa^	(3.4±0.2)^Αb^	(10.3±0.1)^Αb^	(18.4±0.3)^Αb^	(51.8±1.1)^Ab^	(4.5±0.1)^Aa^	(14.6±1.5)^Αa^	(26.3±1.2)^ΑBa^
PP	(64.5±0.3)^Αa^	(3.5±0.1)^Αb^	(10.7±0.2)^Αb^	(18.2±0.1)^Αb^	(52.1±1.4)^Ab^	(4.3±0.1)^Aa^	(14.5±1.1)^Αa^	(26.4±0.3)^Aa^

Values are expressed as mean±standard error. C=control, LA=*L. acidophilus* Alce LMGP21381, LC=*L. casei* 62, LS=*L. sakei* 65, PP=*P. pentosaceus* 156.

Values with different capital letters in superscript indicate statistically significant differences (p*<*0.05) within different treatments on the same day of production.

Values with different lower-case letters in superscript indicate statistically significant differences (p*<*0.05) between the first and the 21st day of production for the same treatment

The total replacement of pork fat with the extra virgin olive oil formulation led to the reduction of fat (14.5-14.8 g/100 g) in the produced fermented sausages (Table 2). This fat reduction was more than 30.0 g/100 g compared to the conventional dry fermented sausages, which ranges between 30.0 and 50.0 g/100 g depending on the country or region of origin (2). This fact could allow the use of the term ‘low fat’ to characterise the fermented sausages produced in this study according to the European Regulation 1924/2006 on health claims (*55*). In the case of US Food and Drug Administration, the term ‘low fat’ has to be used in the statement of identity of a food that bears a relative claim, followed immediately by the name of the nutrient whose content has been altered and a comparative statement (*56*), so ‘low fat, 30% reduced fat content’ could be used.

#### Microbiological parameters

**Fig. 2:**
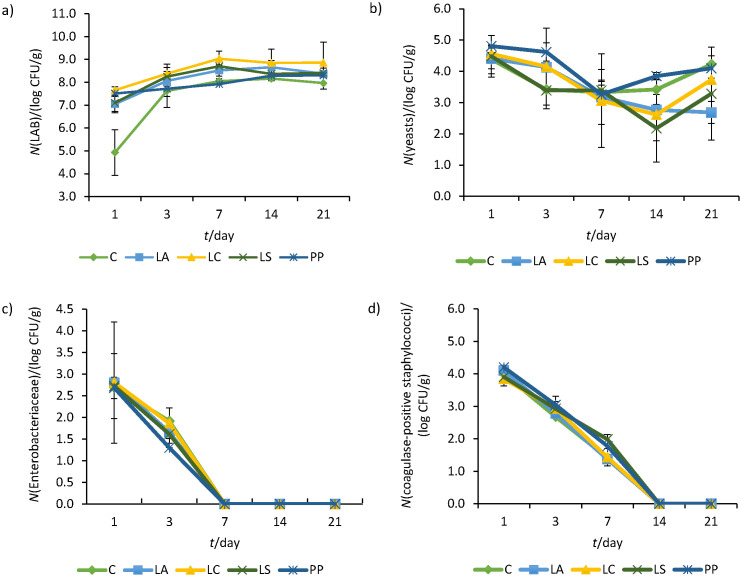
Influence of time on the counts of: a) lactic acid bacteria (LAB), b) yeasts, c) Enterobacteriaceae and d) coagulase-positive staphylococci in dry fermented sausages with extra virgin olive oil and probiotic LAB during production in three independent experiments. C=control, LA=*Lactobacillus acidophilus* Alce LMGP21381, LC=*L. casei* 62, LS=*L. sakei* 65 and PP=*P. pentosaceus* 156. Values are expressed as mean±standard error

The process of fermentation, *i.e*. ripening was completed within 21 days in all treatments. The control samples exhibited significantly lower counts of LAB than the other treatments that included the addition of LAB cultures, although no significant differences were observed among all treatments (Table 3). The counts of the added LAB cultures were approx. 7.0 log CFU/g (Fig. 2) at the beginning of the dry sausage production. The LAB population increased up to the 3rd day, remained stable until the 14th day and displayed a slight decrease on the 21st day of ripening. This reduction, although significant, did not exceed the count of one logarithm. Overall, the LAB populations ranged between 7.5 and 8.5 log CFU/g at the end of the production in all treatments. The fact that the counts of LAB of the controls were similar to those of the other treatments is attributed to the indigenous microbial population of raw materials (meat) consisting of different LAB species and is in accordance with other studies (*45*, *52*). These populations remained stable (ranged between 7.5 and 8.0 log CFU/g) and without significant decrease during the 4th and 6th month of storage (p=0.334-0.531).

**Table 3:** Enterobacteriaceae, staphylococci, lactic acid bacteria (LAB) and yeast counts during fermentation of dry fermented sausages with extra virgin olive oil and probiotic LAB

Microorganism	*t*/day	Treatment				
Control	*L. acidophilus*	*L. casei*	*L. sakei*	*P. pentosaceus*
*N*(microorganism)/(log CFU/g)				
Enterobacteriaceae	1	(2.7±0.8)^Aa^	(2.8±0.8)^Aa^	(2.8±1.4)^Aa^	(2.7±0.6)^Aa^	(2.7±1.3)^Aa^
	3	(1.9±0.2)^Bb^	(1.7±0.2)^Bb^	(1.9±0.4)^Bb^	(1.6±0.2)^Bb^	(1.3±0.2)^Bb^
	7	N.D.^Cc^	N.D.^Cc^	N.D.^Cc^	N.D.^Cc^	N.D.^Cc^
	14	N.D.^Cc^	N.D.^Cc^	N.D.^Cc^	N.D.^Cc^	N.D.^Cc^
	21	N.D.^Cc^	N.D.^Cc^	N.D.^Cc^	N.D.^Cc^	N.D.^Cc^
Staphylococci	1	(4.0±0.2)^Aa^	(4.10±0.06)^Aa^	(3.80±0.01)^Aa^	(3.9±0.2)^Aa^	(4.2±0.0)^Aa^
	3	(2.7±0.2)^Bb^	(2.8±0.3)^Bb^	(2.9±0.0)^Bb^	(2.9±0.0)^Bb^	(3.0±0.2)^Bb^
	7	(1.4±0.3)^Cc^	(1.4±0.3)^Cc^	(1.4±0.2)^Cc^	(2.0±0.2)^Cc^	(1.8±0.1)^Cc^
	14	N.D.^Dd^	N.D.^Dd^	N.D.^Dd^	N.D.^Dd^	N.D.^Dd^
	21	N.D.^Dd^	N.D.^Dd^	N.D.^Dd^	N.D.^Dd^	N.D.^Dd^
Lactic acid bacteria	1	(4.9±1.0)^Aa^	(7.1±0.4)^Bb^	(7.7±0.2)^Bb^	(7.1±1.1)^Bb^	(7.5±0.1)^Bb^
	3	(7.6±0.7)^Ab^	(8.1±0.4)^Bb^	(8.4±0.4)^Bb^	(8.2±0.7)^Bb^	(7.7±0.3)^Ab^
	7	(8.0±0.1)^Ab^	(8.5±0.2)^Bc^	(9.0±0.3)^Bc^	(8.7±0.5)^Bc^	(7.9±0.0)^Ab^
	14	(8.2±0.1)^Ab^	(8.7±0.3)^Ac^	(8.8±0.6)^Ac^	(8.4±0.6)^Ac^	(8.3±0.0)^Ab^
	21	(8.0±0.2)^Ab^	(8.4±0.1)^Ac^	(8.9±0.9)^Ac^	(8.4±0.4)^Ac^	(8.3±0.3)^Ab^
Yeasts	1	(4.4±0.5)^Aa^	(4.4±0.3)^Aa^	(4.6±0.1)^Ab^	(4.5±0.7A)^ab^	(4.8±0.0)^Aa^
	3	(3.4±0.6)^Aa^	(4.1±0.1)^Aa^	(4.2±1.2)^Aa^	(3.4±0.0)^Aa^	(4.6±0.3)^Aa^
	7	(3.3±0.4)^Ab^	(3.2±0.9)^Ab^	(3.1±1.5)^Ab^	(3.4±0.3)^Ab^	(3.3±0.3)^Ab^
	14	(3.4±0.5)^Ab^	(2.8±1.0)^Ab^	(2.6±0.1)^Ab^	(2.2±1.1)^Ab^	(3.9±0.1)^Ab^
	21	(4.2±1.1)^Aa^	(2.7±0.4)^Aa^	(3.7±0.1)^Aa^	(3.3±1.5)^Aa^	(4.1±0.4)^Aa^

Values are expressed as mean±standard error. N.D.=not detected. Values with different capital letters in superscript indicate statistically significant differences (p<0.05) within different treatments on the same day of production. Values with different lower-case letters in superscript indicate statistically significant differences (p<0.05) for the same treatment between various days of fermentation

One of the basic aims of this study was the identification of indigenous LAB cultures with some probiotic properties that would not only be used as starters but could also survive in the final product. According to Ruiz-Moyano *et al*. (*11*), it is critical to ensure the presence of the inoculated culture in the final product in counts more than 6.0 log CFU/g to demonstrate that this culture competes not only with pathogens but with the indigenous microbiota as well, survives in the final product and then is considered to have a potential functional value for the consumer. In this study, at the beginning of the fermented sausage production (day zero), only *L. sakei* was identified in low counts (3.1 log CFU/g; data not shown) in the control, *L. acidophilus* and *L. casei* treatments as part of the indigenous microbiota of the meat, according to the results of the microbiological analysis and the identification of the LAB colonies by real-time PCR. As expected, *L. sakei* population in *L. sakei* treatment on the first day of the production was high (6.9 log CFU/g). *L. casei* and *L. acidophilus* were identified only in the treatments in which they were added (7.6 and 7.0 log CFU/g of *L. casei* and *L. acidophilus*, respectively) on the day of the production. The final LAB counts identified on the 21st day of production by real-time PCR were (in log CFU/g): *L. acidophilus* 5.9, *L. casei* 7.0 and *L. sakei* 5.7 in treatments with *L. acidophilus, L. casei* and *L. sakei*, respectively. The final LAB counts identified after four months of storage by real-time PCR were (in log CFU/g): *L. acidophilus* 5.7, *L. casei* 7.1 and *L. sakei* 5.6 in the respective treatments. Only after six months of storage, the identified LAB by real-time PCR were decreased significantly (in log CFU/g) to: *L. acidophilus* 5.1, *L. casei* 6.7 and *L. sakei* 5.0 in the respective treatments. *P. pentosaceus* was not molecularly identified in the final products since the sausages produced with this isolate obtained low scores in sensory evaluation, as it is described below, and were rejected from further evaluation. Interestingly, *L. casei* 62, isolated from traditional fermented sausages, survived in the highest counts in the final products even after six months of storage, thus, exhibiting better adaptation and competitiveness in the microenvironment of the fermented sausage than the commercial probiotic culture used. Therefore, *L. casei* 62 could be used in large scale production, as it prevailed against other LAB of indigenous microbiota, probably due to its meat-derived origin and survived in higher counts than the commercial starter isolated from milk products. This fact indicates that the isolation and evaluation of autochthonous LAB of meat intended to be used as starters in the production of fermented meat products is quite promising. The need of a variety of starter cultures with probiotic properties is very important as they could provide the meat industry with products with special flavours and organoleptic characteristics in addition to a potential functional value. Various LAB isolates with *in vitro* probiotic properties have been previously isolated from different sources such as fermented milk and meat (*9*-*11*). Some of them could be promising candidates for further investigation such as the *L. casei* 62 isolated in the present study. Nevertheless, the evaluation of the *in vitro* probiotic properties of such LAB isolates should be combined with their practical use in fermented sausage production in order to evaluate their technological characteristics as well.

Consumers do not prefer dry fermented sausages for their functional value, as they tend to do for dairy products. In this study, a novel and premium meat product was produced with extra virgin olive oil and LAB that exhibited some probiotic properties *in vitro*. On the other hand, any reference to ‘probiotic properties’ as a health claim cannot appear on any food unless results from specific clinical studies support such a function beyond any doubt (*8*, *55*). However, the isolate *L. casei* 62 proved to be a promising LAB isolate since it exhibited some probiotic properties *in vitro* and its further investigation including clinical studies could prove beneficial in order to prove any *in vivo* functional value. This isolate in combination with the reduction of fat content and desirable alteration of the lipid acid profile, due to the presence of the monounsaturated fatty acids of extra virgin olive oil origin, could offer the meat industry a new premium dry fermented meat product with nutritional benefits for the consumers.

Apart from LAB, the population of yeasts gradually decreased until the 7th day and increased afterwards. *L. monocytogenes* was not detected and moulds were below the limit of detection regarding all treatments. Enterobacteriaceae and coagulase-positive staphylococci counts were not affected significantly by the addition of LAB cultures (p=0.393-0.954) and did not survive in any treatment after the 7th and 14th day, respectively. In previous studies, staphylococci have been detected in the final products of fermented sausages, thus, raising food safety apprehensions (*57*). A decrease to non-detectable counts of both Enterobacteriaceae and staphylococci was observed in this study, an indicative fact of the high quality of raw materials, hygienic handling and the effective addition of LAB as starter cultures.

#### Sensory properties of sausages

**Fig. 3:**
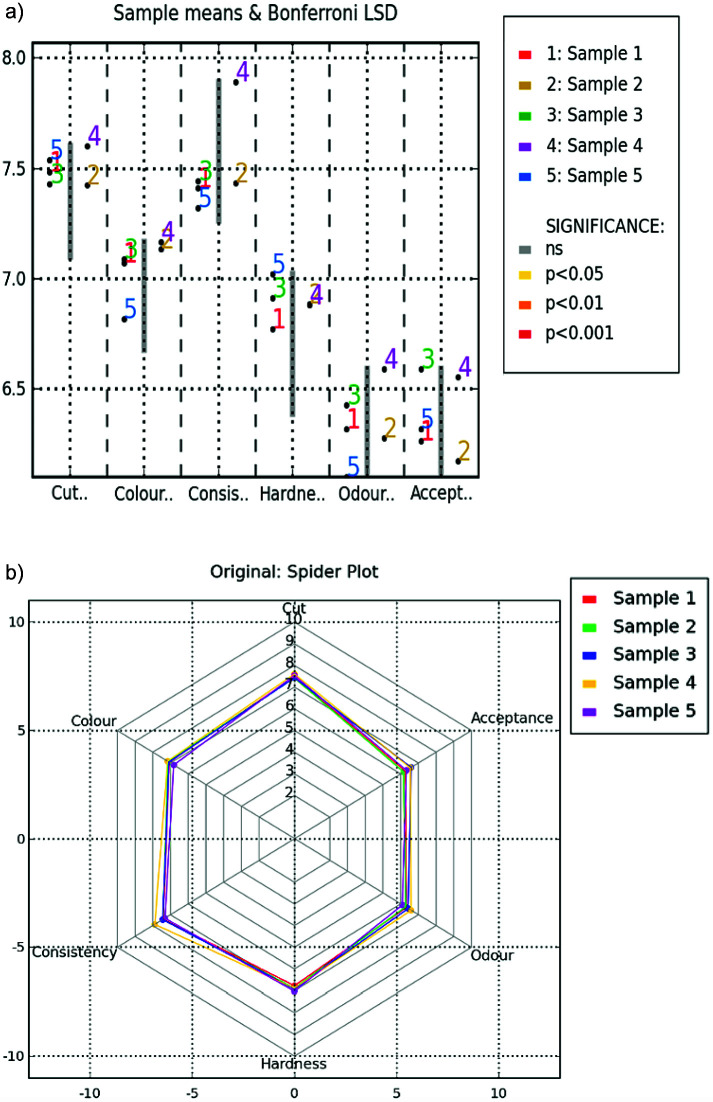
Sensory evaluation profiling (appearance of the cut, colour, cohesiveness, hardness, flavour, overall acceptance) of dry fermented sausages with extra virgin olive oil and probiotic lactic acid bacteria (LAB) on the 21st day of production in three independent experiments presented as: a) sample mean values with Bonferroni's least significance difference (LSD) test and b) spider plot. 1=control, 2=*L. acidophilus* Alce LMGP21381, 3=*L. casei* 62, 4=*L. sakei* 65, 5=*P. pentosaceus* 156

The addition of different LAB did not affect significantly the appearance of the cut surface (p=0.912), colour (p=0.852), consistency (p=0.963) and hardness (p=0.664) among the different treatments. The taste/odour (p=0.886), which are affected by the provoked lipolysis and proteolysis by the starter culture (*58*) and the overall acceptability (p=0.880), were not affected significantly among the different treatments (Fig. 3). All LAB isolates used in this study belong to the species commonly used in fermented products. Nevertheless, the desirable sensory characteristics attributed to the final products depend on the isolate used in each case (*59*).

The olive oil-based fat substitute formed a mosaic pattern that was visible in the sliced final product and very similar in appearance to the visible fat in conventional fermented sausages. Moreover, this olive oil-based formulation replaced totally the added pork fat and not partially as in previous studies, where sausages with partial fat replacement displayed appearance or texture deficiencies compared to the conventional dry fermented sausages (*2*, *5*, *6*, *60*). However, total replacement of the added pork fat by a so-called konjac matrix containing different seed and fish oils has been previously achieved (*2*), but the final products displayed undesirable sensory properties regarding hardness, appearance and overall acceptance in contrast to the fermented sausages produced in the present study. Therefore, the olive oil-based formulation designed in this study is regarded an ideal fat substitute for the production of fermented sausages.

#### Textural and colour characteristics

The textural parameters of produced sausages are presented in Table 4. Fat replacement with olive oil emulsions affected significantly (p<0.001) all textural properties except cohesiveness (p=0.350). Treatments with emulsions had lower values of springiness, gumminess and chewiness than the control treatment. Moreover, olive oil emulsions increased textural properties like hardness, chewiness and cut test measurements. These objective measurements are in agreement with the sensory evaluation by experienced panellists as described previously. Colour is regarded as the most important sensory attribute οf meat products by consumers (*61*). The addition of different LAB did not significantly affect the colour of the samples (p=0.330), but all treatments resulted in higher scores of lightness and redness than the control (p<0.001) (Table 5).

**Table 4:** Textural properties of fermented sausages produced with pasteurised olive oil emulsions

Sample	Texture profile analysis								Cut test	
Hardness F_1_/N	A_1_/J	Springiness S_2_/m	Hardness F_2_/N	A_2_/J	CohesivenessΑ_2_/Α_1_	Gumminess/N	Chewiness/(N/cm)	A/J	F/N
C	(500±58)^C^	(2.3±0.2)^B^	(0.036±0.002)^C^	(304±60)^B^	(0.6±0.1)^B^	(0.21±0.01)^A^	(103.9±8.7)^B^	(3.7±0.2)^B^	(0.61±0.01)^B^	(20.7±1.3)^B^
LA	(753±29)^B^	(2.90±0.01)^AB^	(0.040±0.004)^B^	(559±59)^A^	(0.7±0.1)^A^	(0.23±0.02)^A^	(170.4±18.9)^A^	(6.8±0.7)^A^	(1.38±0.03)^AB^	(47.3±4.1)^A^
LC	(860±28)^AB^	(3.2±0.8)^A^	(0.040±0.003)^B^	(596±32)^A^	(0.7±0.1)^A^	(0.21±0.02)^A^	(177.2±17.8)^A^	(7.1±0.7)^A^	(1.5±0.1)^AB^	(47.7±13.2)^A^
LS	(898±66)^AB^	(3.2±0.3)^A^	(0.040±0.001)^B^	(620±63)^A^	(0.7±0.1)^A^	(0.21±0.03)^A^	(191.5±20.6)^A^	(7.6±0.8)^A^	(1.6±0.3)^A^	(43.6±2.6)^A^
PP	(989±73)^A^	(3.4±0.3)^A^	(0.040±0.004)^A^	(682±70)^A^	(0.7±0.1)^A^	(0.21±0.01)^A^	(204.4±15.6)^A^	(9.0±0.7)^A^	(1.2±0.4)^AB^	(35.3±4.8)^A^

C=control, LA=*L. acidophilus* Alce LMGP21381, LC=*L. casei* 62, LS=*L. sakei* 65, PP=*P. pentosaceus* 156, F_1_ and F_2_=force of cut during the first (A_1_) and the second bite (A_2_). Values with different capital letters in superscript indicate statistically significant differences (p*<*0.05) within different treatments of the final products

**Table 5:** Colour parameters of fermented sausages produced with olive oil emulsions

Treatment	Colour		
L*	a*	b*
C	(45.2±1.3)^B^	(13.3±0.5)^A^	(3.1±0.5)^B^
LA	(46.3±2.1)^A^	(13.4±0.4)^A^	(5.5±0.4)^A^
LC	(46.6±1.6)^A^	(13.2±0.8)^A^	(5.1±0.6)^A^
LS	(46.2±1.4)^A^	(13.5±0.9)^A^	(5.2±0.5)^A^
PP	(46.8±1.9)^A^	(13.3±0.3)^A^	(5.3±0.6)^A^

L*=lightness, a*=redness, b*=yellowness, C=control, LA=*L. acidophilus* Alce LMGP21381, LC=*L. casei* 62, LS=*L. sakei* 65, PP=*P. pentosaceus* 156.

Values with different capital letters in superscript indicate statistically significant differences (p*<*0.05) within different treatments of the final products

## Conclusions

In the present study, a novel and high-quality dry fermented meat product was produced. This product managed to combine the total replacement of added pork fat by an extra virgin olive oil emulsion with turkey meat along with the addition of LAB cultures, exhibiting some probiotic properties *in vitro*, isolated from the indigenous microbiota of traditional fermented meat products. The olive oil formulation generated a visible mosaic pattern in the sliced final product that simulated successfully the visible pork fat in the conventional fermented sausages. After fermentation and during sensory evaluation, the produced sausages obtained high scores in all tested parameters such as flavour, firmness, appearance and overall acceptance. Autochthonous LAB with *in vitro* probiotic properties could have a potential use in large-scale novel dry fermented sausage manufacture. Such isolates could be used as starters in an effort to standardise the production process and retain the typical organoleptic and sensory characteristics. Moreover, isolates like *L. casei* 62 that survived in high counts in the final products in this study could increase the safety of fermented sausages by competing not only with pathogens but also with the indigenous microbiota and could exhibit a potential functional value for the consumers.
